# Exploring strategies to reach individuals of Turkish and Moroccan origin for health checks and lifestyle advice: a mixed-methods study

**DOI:** 10.1186/s12875-016-0476-1

**Published:** 2016-07-21

**Authors:** Andrea J. Bukman, Dorit Teuscher, Jamila Ben Meftah, Iris Groenenberg, Mathilde R. Crone, Sandra van Dijk, Marieke B. Bos, Edith J. M. Feskens

**Affiliations:** Division of Human Nutrition, Wageningen University, P.O Box 8129, 6700 EV Wageningen, The Netherlands; Department of Human Biology, Maastricht University Medical Centre+, NUTRIM School for Nutrition, Toxicology and Metabolism, P.O Box 616, 6200 MD Maastricht, The Netherlands; Department of Public Health and Primary Care, Leiden University Medical Center, Hippocratespad 21, 2300 RC Leiden, The Netherlands; Health, Medical and Neuropsychology Unit, Faculty of Social and Behavioral Sciences, Leiden University, P.O Box 9555, 2300 RB Leiden, The Netherlands; Dutch Heart Foundation, P.O Box 300, 2501 CH The Hague, The Netherlands

**Keywords:** Mixed-methods, Ethnic groups, Health check, Lifestyle advice, Reach

## Abstract

**Background:**

Low participation rates among ethnic minorities in preventive healthcare services are worrisome and not well understood. The objective of this study was to explore how adults of Turkish and Moroccan origin living in the Netherlands, aged 45 years and older, can be reached to participate in health checks for cardio-metabolic diseases and follow-up (lifestyle) advice.

**Methods:**

This mixed-methods study used a convergent parallel design, to combine data of one quantitative study and three qualitative studies. Questionnaire data were included of 310 respondents, and interview data from 22 focus groups and four individual interviews. Participants were recruited via a research database, general practitioners and key figures. Quantitative data were analysed descriptively and qualitative data were analysed using a thematic approach.

**Results:**

Regarding health checks, 50 % (95 % CI 41;59) of the Turkish questionnaire respondents and 66 % (95 % CI 57;76) of the Moroccan questionnaire respondents preferred an invitation from their general practitioner. The preferred location to fill out the health check questionnaire was for both ethnic groups the general practitioner’s office or at home, on paper. Regarding advice, both groups preferred to receive advice at individual level rather than in a group, via either a physician or a specialised healthcare professional. It was emphasised that the person who gives lifestyle advice should be familiar with the (eating) habits of the targeted individual. Sixty-one percent (95 % CI 53;69) of the Turkish respondents preferred to receive information in their native language compared to 37 % (95 % CI 29;45) of the Moroccan respondents. Several participants mentioned a low proficiency in the local language as an explanation for their preference to fill out the health check questionnaire at home, to receive advice from an ethnic-matched professional, and to receive information in their native language.

**Conclusions:**

The general practitioner is considered as a promising contact to reach adults of Turkish and Moroccan origin for health checks or (lifestyle) advice. It might be necessary to provide information in individuals’ native language to overcome language barriers. In addition, (lifestyle) advice must be tailored. The obtained insight into preferences of Turkish and Moroccan adults regarding reach for preventive healthcare services could help professionals to successfully target these groups.

## Background

Cardio-metabolic diseases, like cardiovascular diseases and diabetes mellitus, have a substantial impact on the global burden of disease [1, 2]. The risk for developing cardio-metabolic diseases seems to be especially high among some ethnic minorities [3–5].

In the Netherlands, as in several other European countries, individuals of Turkish and Moroccan origin are the two largest non-Western ethnic minority groups [6]. Among these ethnic minorities, the prevalence of type 2 diabetes is relatively high compared to the host population [7–9]. There is also evidence for relatively unfavourable HDL cholesterol levels among people of Turkish origin [8].

An increased risk of cardio-metabolic diseases can be due partly to modifiable risk factors, such as an unhealthy lifestyle and overweight [10, 11]. In the Netherlands, individuals of Turkish and Moroccan origin have a relatively high prevalence of physical inactivity and overweight compared to individuals of Dutch origin [12, 13]. Given their elevated risk, these groups in particular could benefit from preventive health services.

However, it seems difficult to reach ethnic minorities for preventive health services. The lack of reach hinders both the identification of individuals at high risk and the subsequent uptake of health promoting activities. Firstly, concerning identification, ethnic minorities are often not reached for health screening services [14–17]. This poses a problem, as early detection of individuals at risk for metabolic diseases is of utmost importance in order to prevent health complications and to offer lifestyle advice to those who need it. Secondly, ethnic minorities are less likely to be reached by health promoting activities [18, 19]. However, the problem does not seem to be that ethnic minorities do not have access to healthcare, as in the Netherlands they often visit their GP [20]. Still, there is a lack of specific strategies to reach individuals of Turkish and Moroccan origin for preventive health services.

Recently, a health check for cardio-metabolic diseases was developed in the Netherlands. This health check consists of a two-stage approach. In the first stage, people fill out a short questionnaire (risk estimation). In the second stage, people with a high risk score are advised to plan two consultations with their GP or practice nurse for further examination of their risk profile and to discuss follow-up actions [21]. Along with the development of this health check, the issue was raised how to reach individuals of Turkish and Moroccan origin for this initiative and how to provide suitable follow-up lifestyle advice. To solve this issue, several studies were initiated. The ambition of this paper was to combine insights of these independent studies, which included one quantitative study and three qualitative studies.

The overall aim of the current study was to get insight into the perceptions of adults of Turkish and Moroccan origin living in the Netherlands regarding how they could successfully be reached for both a health check for cardio-metabolic diseases and follow-up (lifestyle) advice. To this end, this study provided answers to the following questions among the two ethnic groups:

- By whom do they prefer to be invited for a health check, and why?
- Where do they prefer to fill out a health check questionnaire, and why?
- By whom do they want to receive (lifestyle)advice, and why?
- What is the preferred way of communicating (lifestyle)advice, and why?
- What is the preference regarding language, and why?

## Methods

The current study is a secondary analysis, using data from four related studies (one quantitative study and three qualitative studies), among adults of Turkish and Moroccan origin living in the Netherlands. To answer the research questions of the current study, a selection of data of the four studies were used. The relation between the original studies and the data used in the current study is presented in Table 1. The studies were conducted independently of one another and all provided data regarding either participating in a health check or receiving (lifestyle) advice, or both. A mixed-methods approach – “in which elements of quantitative and qualitative research approaches are combined” [22] – was used in order to get a better understanding of the quantitative results regarding the research questions of the current study, with the help of the narratives from the qualitative studies.

**Table 1:** Overview of objectives and methods of the original studies and data used in current study

	Original study objective	Original study population	Data collection	Recruitment strategy	Data included in secondary analysis	Relevant topics for secondary analysis
*Quantitative study*	To get insight into knowledge and perceptions of adults of Turkish and Moroccan origin regarding cardiovascular diseases and its risk factors, and their preferences in order to reach them with health communication.	Individuals of Turkish and Moroccan origin (18 years and older)	Web-based questionnaire	Via TNS NIPObase (database for market research)	310 respondents, aged 45 and older: • 167 Turks • 143 Moroccans	Health check: • by whom? • where? CVD information: • by whom? • preferred way? • preferred language?
			Face-to-face interviews, using a structured questionnaire	Via research assistants in own network		
*Qualitative study I*	To explore determinants influencing vulnerable groups regarding (non-) participation in the Dutch two-stage cardiometabolic health check, comprising a health risk assessment and prevention consultations for high-risk individuals.	Non-Western immigrants and Dutch individuals with low socioeconomic status (45–70 years old)	Focus groups, using a semi-structured interview guide	Via key persons within the community, e.g. educational coordinators or employees of cultural/community organisations	4 focus groups: • 2 Turkish groups • 2 Moroccan groups	Health check: • by whom? • where? • preferred language?
		Adult children of the non-Western immigrants (18–45 years old)	Focus groups, using a semi-structured interview guide		5 focus groups: • 3 Turkish groups • 2 Moroccan groups	
*Qualitative study II*	To explore factors that play a key role in the uptake and maintenance of behavioural changes in individuals from non-western immigrant populations with a high risk for cardiometabolic disease. Furthermore to get insight in what kind of support is needed to increase the uptake and maintenance of healthy behaviours.	Non-Western immigrants at risk for cardio-metabolic diseases (45–70 years old)	Face-to-face interviews, using a semi-structured interview guide	Via their GP	4 face-to-face interviews: • 2 Turks • 2 Moroccans	Lifestyle advice: • by whom? • preferred way? • preferred language?
		Adult children of the non-Western immigrants (18–45 years old)	Focus groups, using a semi-structured interview guide	Via community workers and neighbourhood centres	3 focus groups: • 1 Turkish group • 2 Moroccan groups	
*Qualitative study III*	To explore perceptions on healthy eating and physical activity of individuals with lower socioeconomic status of different ethnic origin, in order to identify opportunities to make a lifestyle intervention more applicable to the target groups’ realities.	Individuals of Turkish, Moroccan and Dutch origin with low socioeconomic status (45 years and older)	Focus groups, using a semi-structured interview guide	Via local community workers, chairmen of mosques and persons of the target population	10 focus groups: • 6 Turkish groups • 4 Moroccan groups	Lifestyle advice: • by whom? • preferred way? • preferred language?

### Study population and data collection

The demographic characteristics of the study population included in the current mixed method study are presented in Table 2. Study population, study procedure, and the data collection methods for each original study are described below. Additionally, for each study, it is described which data is used in the current mixed methods study.

**Table 2:** Characteristics of the participants in the four individual studies in this mixed methods study

	Quantitative study		Qualitative study I		Qualitative study II		Qualitative study III	
	Turks	Moroccans	Turks	Moroccans	Turks	Moroccans	Turks	Moroccans
Target group	*n* = 167	*n* = 143	*n* = 15	*n* = 18	*n* = 2	*n* = 2	*n* = 33	*n* = 33
Gender								
Males Females	51 % 49 %	73 % 27 %	1 group 1 group	1 group 1 group	– 2 interviews	– 2 interviews	3 groups 3 groups	2 groups 2 groups
Age (mean years ± SD)	53 ± 8.1	55 ± 8.7	52 ± 8.5	54 ± 6.8	55 ± 3.5	48 ± 2.8	49 ± 8.5	47 ± 11.8
Overweight (BMI > 25 kg/m^3^)	82 %	73 %	–	–	–	–	85 %	87 %
Adult children			*n* = 22	*n* = 10	*n* = 6	*n* = 13		
Gender								
Males Females Mixed	– – –	– – –	1 group 2 groups –	– 2 groups –	– – 1 group	1 group 1 group –	– – –	– – –
Age (mean years ± SD)	–	–	34 ± 13.4	19 ± 3.6	31 ± 12.2	28 ± 7.7	–	–

#### Quantitative study

##### Study population

The target group of the original study consisted of adults of Turkish and Moroccan origin aged 18 years and older.

##### Procedure

A representative sample design was composed, based on the background characteristics of the target group (for each ethnic group: by region, gender, age, education) by using data from Statistics Netherlands. On the basis of this design, 600 persons were selected from the TNS NIPObase, which is a database for market research. Selected persons received an invitation by e-mail to fill out a web-based questionnaire. The persons had two and a half weeks to fill in the questionnaire. A reminder was sent after one and a half weeks. The response rate was 52 % (*N* = 313). An additional sample was recruited for face-to-face interviews (*N* = 586). These interviews were conducted in Dutch, using a structured questionnaire similar to the web-based questionnaire. The interviews were held by specialized personnel of a market research agency. The web-based questionnaire was distributed in March 2010 and the face-to-face interviews were conducted between April and June 2010. The research team assessed that, according to the Dutch regulations, no ethical permission was required for this type of research [23].

##### Questionnaire

The questionnaire consisted of 74 questions divided over four topics, namely, questions regarding participants’: 1) general characteristics, 2) health and lifestyle, 3) knowledge and attitude towards cardiovascular diseases (CVD) and health checks, and 4) preferences regarding information provision concerning CVD. It was aimed to construct a questionnaire that would take 30 min to fill in. The questionnaire was self-constructed by a market research agency specialised in the target population. The questionnaire was pre-tested for duration and clarity among eight subjects (4 of Turkish origin and 4 of Moroccan origin) and was adjusted based on the findings of the pre-test.

##### Data used in secondary analysis

For the mixed methods study, data were used of respondents aged 45 years and older, of whom 167 were of Turkish origin and 143 were of Moroccan origin. Data were only used from those questions related to reach for a health check or related to information provision concerning CVD (Table 3). It should be noted that questions regarding a health check were examined only among those respondents that were either ‘maybe’ or ‘definitely’ interested in participating in a health check (85 % of the Turkish respondents and 71 % of the Moroccan respondents).

**Table 3:** Selected questions from quantitative study

A health check:	Information concerning CVD:
• In the future, a new health check will be provided that is scientifically tested. The check starts with a questionnaire. From your answers, it may emerge, for example, that you have an elevated risk of getting diabetes and/or cardiovascular diseases. If so, then you will be advised to visit your GP for further investigation. Would you like to participate in this new health check? *□ Yes, definitely* *□ Yes, maybe* *□ No, probably not* *□ No, definitely not* If ‘*Yes, definitely*’ or ‘*Yes, maybe*’: • By whom would you prefer to be invited for this check? *□ GP* *□ Specialist/hospital* *□ Municipal institution/Community health service* *□ Other* *□ Don’t know* • Where would you like to fill out this questionnaire? *□ At GP’s office* *□ At the specialist’s/in the hospital* *□ At community health service* *□ At other medical healthcare provider* *□ In the community centre* *□ In the mosque* *□ At home, with pen and paper* *□ Via internet* *□ Other* *□ No preference*	• Suppose that you are interested in information about CVD, where would you get that information? Mention two most important information sources. *□ GP* *□ Specialist/hospital* *□ Community health service* *□ Dutch Heart Foundation* *□ Internet* *□ Library* *□ Other* • What is the preferred way of communicating information about CVD? *□* W*ritten via brochure/paper* *□ Internet* *□ Oral in a group* *□ Oral in person* *□ Television* *□ Other* • Do you want to receive the information about CVD in Dutch or your native language? *□ Prefer Dutch* *□ Prefer native language* *□ Does not matter* • Suppose that a person provides information about CVD.Do you consider it important that this person: …is a physician/doctor? …is of the same ethnic origin? …is of the same gender? *□ Very important* *□ A little important* *□ Not important* *□ Not important at all*

#### Qualitative study I

##### Study population

The target group of this study consisted of non-Western immigrants and native Dutch participants with a low socioeconomic status (45–70 years old). Although the target group consisted of persons aged 45–70 years, adult children of the non-Western immigrants (18–45 years old) were also invited for this study. This was done to overcome language barriers and because these children often help their parents to find their way around the Dutch healthcare system. Adult children were interviewed about the needs and preferences of their parents. The methods used in this study are presented in detail elsewhere [24].

##### Procedure

Participants were recruited through key persons within the community, e.g. educational coordinators or employees of cultural/community organisations. The focus groups were conducted between February and June 2010. They were held separately for males and females. The focus groups were held in Dutch by the researcher (IG), who was accompanied by an ethnic matched research assistant who took notes and helped the interviewer, if the mastery of the Dutch language of participants was low. The study was approved by the medical ethics committee of Leiden University Medical Center. Participants’ verbal informed consent was audio-taped. The interviews were recorded and transcribed verbatim. Interviews transcripts were coded with Atlas.ti 6.2.

##### Interview guideline

A semi-structured interview guide was used, with questions regarding invitation strategies, risk communication and determinants influencing participation in health checks.

##### Data used for secondary analysis

For the mixed methods study, data were used from four focus groups with the target group (2 Turkish groups; 2 Moroccan groups) and five focus groups with the adult children of the target group (3 Turkish groups; 2 Moroccan groups).

#### Qualitative study II

##### Study population

The target group of this study consisted of non-Western immigrants (45–70 years old) at risk for cardio-metabolic diseases. Like in qualitative study I, adult children of non-Western immigrants (18–45 years old) were also included in the study.

##### Procedure

The older adults were recruited via their GP, and adult children were recruited via community workers and neighbourhood centres. The (focus group) interviews were conducted between February 2011 and January 2012. Focus groups were organised for men and women separately, if the participants preferred that over a mixed group. The (focus group) interviews were held in Dutch by the researcher (JBM). During the focus groups, the researcher was accompanied by a research assistant who took notes and who was ethnically matched if translating was needed. Likewise, if necessary, a translator was present during the face-to-face interviews. The study was approved by the medical ethics committee of Leiden University Medical Center. Participants signed an informed consent form. The interviews were recorded and transcribed verbatim. Interviews transcripts were coded with Atlas.ti 6.2.

##### Interview guideline

A semi-structured interview guide was used, with questions regarding culture and health, the uptake and maintenance of healthy behaviours, and preferences regarding lifestyle guidance.

##### Data used for secondary analysis

For the mixed methods study, data were available from four face-to-face interviews with the target group (2 Turks; 2 Moroccans) and three focus groups with the adult children of the target group (1 Turkish group; 2 Moroccan groups).

#### Qualitative study III

##### Study population

The target group of this study consisted of native Dutch participants and participants of Turkish and Moroccan origin, aged 45 years and older. The methods used in the original study are presented in detail elsewhere [25, 26].

##### Procedure

The focus groups were conducted between May and November 2011 and were held separately for males and females. Participants were recruited via local community workers, chairmen of mosques and persons of the target population in mostly disadvantaged neighbourhoods. The focus groups were held in Dutch by one of the two researchers (AJB and DT) and the other researcher took notes. If participants could not express their feelings in Dutch, they expressed themselves in their native language and others translated for the researchers. The study was approved by the medical ethics committee of academic hospital Maastricht and Maastricht University (METC azM/UM). Participants gave either written or audio-taped informed consent. The interviews were recorded and transcribed verbatim. Interviews transcripts were coded with Nvivo 9.2.

##### Interview guideline

A semi-structured interview guide was used with questions regarding determinants of a healthy diet and physical activity, and experiences and preferences regarding lifestyle guidance.

##### Data used for secondary analysis

For the mixed methods study, data were used from ten focus groups with the target group (6 Turkish groups; 4 Moroccan groups).

### Mixed-methods analysis

For the secondary data analyses, a mixed-methods approach was used with a convergent parallel design. This mixed-methods study had a quantitative priority, meaning that greater emphasis was placed on the quantitative findings for answering the study question. The qualitative data were used to explain and elaborate on the quantitative findings.

Firstly, the data of the four studies were prepared (e.g. transcribed, coded) independently by each research team that conducted the original study. Secondly, the first author (AJB) studied the quantitative data. Quantitative data were analysed with SPSS Statistics 22. Data were described using frequency tables. A bootstrap analysis with 1000 simulations was used to calculate 95 % confidence intervals. Data were compared between participants of Turkish and Moroccan origin using Chi-Square tests. Thirdly, the three research teams of the qualitative studies I, II & III used their qualitative data to explain the findings of the quantitative data. Finally, the first two authors (AJB and DT) compared and combined the results of the previous step. During this process, the original data were consulted when necessary. The final results were checked by all research teams to ensure that no information was missed or misinterpreted.

## Results

### Health check

Regarding reach for a health check, the following topics were examined: preferred source of invitation and preferred location to fill out a health check questionnaire.

#### Source of invitation

As described in Table 4, most of the questionnaire respondents would prefer to be invited for a health check by their GP (50 % (95 % CI 41;59) of the Turkish respondents and 66 % (95 % CI 57;76) of the Moroccan respondents). Several participants in the qualitative studies explained that they trust their GP and take it seriously when a GP sends them something. They indicated that, if they were invited by the GP, they would participate.

“If you receive it from the GP, you will fill it out, I’m sure of that. Because if it is from the GP, they will think: Yes.” (Turkish female adult child)

**Table 4:** Preferences regarding source of invitation and location to fill out a health check questionnaire. The table presents the percentage (with 95 % confidence interval) of participants that chose that option; one option should be chosen

	Turks (*n* = 142)	Moroccans (*n* = 102)	p*
By whom would participants like to be invited for a health check			
GP	50 % (41; 59)	66 % (57; 76)	0.001^a^
Specialist/hospital	41 % (32; 50)	18 % (10; 25)	
Municipal institution/Community health service	2 % (0; 5)	7 % (2; 12)	
Other	0 %	5 % (1;10)	
Don’t know	7 % (3; 12)	5 % (1;9)	
Preferred location to fill out a health check questionnaire			
At GP’s office	40 % (32; 48)	39 % (30; 49)	0.48^b^
At home, with pen and paper	23 % (16; 29)	23 % (15; 30)	
At the specialist’s/in the hospital	20 % (14; 27)	13 % (6; 19)	
Via internet	8 % (4; 14)	9 % (4; 15)	
In the mosque	2 % (0; 5)	2 % (0; 5)	
In the community centre	1 % (0; 4)	0 %	
At community health service	0 %	4 % (1; 8)	
At other medical healthcare provider	0 %	0 %	
Other	1 % (0; 2)	2 % (0; 5)	
No preference	5 % (1; 9)	9 % (3; 15)	

* p-value of Pearson Chi-Square tests

^a^The categories ‘Other’ and ‘ Don’t know’ were combined in order to comply to the assumptions of the Pearson Chi-Square test

^b^The categories ‘In the mosque’, ‘In the community centre’, ‘At community health service’, ‘At other medical healthcare provider’ and ‘Other’ were combined to comply to the assumptions of the Pearson Chi-Square test

However, not everyone trusts their GP. Some participants indicated that they do not have the feeling that their health complaints are taken seriously.

“When you are at the GP, she already starts writing: paracetamol, while you’re telling your story.” (Turkish male)

“They send us home with a paracetamol, while it *[the complaint]* is really more severe. They *[my parents]* won’t be taken seriously.” (Moroccan female adult child)

Forty-one percent (95 % CI 32;50) of the respondents of Turkish origin indicated medical specialists/the hospital as their preferred source to invite them for a health check, compared to 18 % (95 % CI 10;25) of the respondents of Moroccan origin. An invitation by a specialist or hospital was not extensively discussed in the qualitative studies. If the invitation was from the hospital, some participants stated that it would be important that they were familiar with that hospital.

“They have to be familiar with it. If it comes from a hospital that they don’t know, I would also say: ‘What do you want from me’. You put it *[the invitation]* away and you forget about it.” (Turkish female adult child)

#### Preferred location to fill out the health check questionnaire

Most respondents in the quantitative study indicated that they would prefer to fill out a health check questionnaire at the GP’s office (40 % (95 % CI 32;48) of the Turkish respondents and 39 % (95 % CI 30;49) of the Moroccan respondents). Participants in the qualitative studies explained that, at the GP’s office, the GP could tell them about the test and give more information when necessary. Another reason to fill out the questionnaire at the GP’s office is because they have time anyway, while waiting for their appointment.

“In the waiting room, persons are bored anyway. The GP can provide the questionnaires in the waiting room. Those persons can fill out the questionnaire on site.” (Turkish female adult child)

The preference for filling out the health check questionnaire at home, on paper, was mentioned by 23 % (95 %CI 16;29) of the Turkish questionnaire respondents and 23 % (95 % CI 15;30) of the Moroccan respondents. Participants in the qualitative studies explained that the advantage of receiving a letter at home was that they could take the time to read and understand the letter, or they could ask someone else to translate it for them.

“Personally I prefer a letter. It is better for people who do not speak Dutch. Why? Because if they receive a letter, they will think: ‘Oh, I received a letter so I will go to my cousin who does speak Dutch and he can read and translate the letter for me’. They will understand the message better.” (Turkish male adult child)

Some participants in the qualitative studies suggested providing the questionnaire in their mother tongue – possibly in addition to the Dutch version – to be able to understand it themselves and not to be dependent on others for a translation. However, even if the questionnaire is provided in the person’s mother tongue, some persons might not be able to read it.

“When I look at my own situation, it does not add any value if it is in Arabic, because my mother is illiterate.” (Moroccan female adult child)

### Advice

Regarding reach for follow-up (lifestyle) advice, the following topics were examined: preferred way to receive advice, source of advice and language. It should be noted that the quantitative findings refer to advice regarding CVD in general, whereas the qualitative findings refer to advice regarding lifestyle specifically.

#### Preferred way to receive advice

As mentioned by 65 % (95 % CI 57;72) of the Turkish respondents and 51 % (95 % CI 42;60) of the Moroccan respondents, the preferred way to receive information about CVD was in person, on an individual level (see Table 5). In one of the qualitative studies, it was asked how participants would prefer to receive nutrition and physical activity advice. The answers depended on the type of information that would be expected; in the case of personal information, some participants in the qualitative studies expressed a preference for advice on an individual level because not everyone should know about their personal eating habits:

“If you talk about my lifestyle in particular, yes, then it is nice of course *[to discuss it on an individual level]*. Otherwise, everyone knows ‘oh, he has such a belly, because he eats that and that’. That is not pleasant of course. That advice, when it is about changing my lifestyle for example, then not everyone has to know that.” (Moroccan male)

However, in the case of general information, group meetings were appreciated. Participants indicated that receiving general advice in group meetings was better because they could stimulate and support one another.

**Table 5:** Preferences regarding way of communicating CVD advice. The table presents the percentage (with 95 % confidence interval) of participants that chose that option; multiple options were possible

	Turks (*n* = 167)	Moroccans (*n* = 143)	p*
Where would you get information			
GP	78 % (72; 85)	76 % (70; 82)	0.68
Specialist/hospital	44 % (37; 52)	40 % (31; 47)	0.49
Internet	23 % (17; 29)	17 % (11; 24)	0.26
Dutch Heart Foundation	11 % (6; 16)	8 % (5; 13)	0.56
Community health service	7 % (4; 12)	6 % (2; 9)	0.65
Library	1 % (0; 3)	1 % (0; 4)	1.00
Other	4 % (1; 7)	14 % (9; 20)	0.004
Preferred way of receiving information			
Oral in person	65 % (57; 72)	51 % (42; 60)	0.021
Written via brochure/paper	39 % (32; 47)	43 % (36; 51)	0.49
Oral in a group	21 % (15; 27)	12 % (7; 17)	0.034
Internet	19 % (13; 25)	23 % (16; 30)	0.40
Television	13 % (8; 18)	12 % (7; 17)	1.00
Other	1 % (0; 2)	5 % (2; 9)	0.026

* p-value of Fisher’s Exact tests

#### Source of advice

**Fig. 1:**
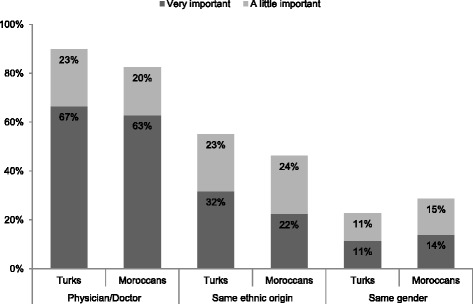
Preferences regarding profession, ethnicity and gender of the person that provides information about CVD. The figure presents the percentage of Turkish and Moroccan participants that considered it either ‘very important’ or ‘a little important’ that the person that provides information is a physician/doctor, of the same ethnic origin and of the same gender. The rest of the participants considered these characteristics of the advisor ‘not important’ or ‘not important at all’

When asked for the two most important sources for information about CVD, most of the respondents indicated that they would get their information from their GP (78 % (95 % CI 72;85) of the Turkish respondents and 76 % (95 % CI 70;82) of the Moroccan respondents) or a medical specialist/hospital (44 % (95 % CI 37;52) and 40 % (95 % CI 31;47), respectively) (see Table 5). To most of the respondents, it is a little to very important that the person who gives advice is a physician/doctor (90 % (95 % CI 85;94) and 83 % (95 % CI 77;89) for Turks and Moroccans, respectively) (see Fig. 1). For the participants in the qualitative studies, it was especially important that the person giving advice was competent. Specialised health professionals were suggested, such as a dietician for nutrition advice.

“Preferably someone who knows a lot about that, someone who’s professional in that field.” (Turkish female)

Furthermore, it was emphasised that the person who gives lifestyle advice should be familiar with the eating habits of the targeted individual.

“But the person has to have knowledge about our dietary habits, what we eat and so on. Because if the advice is like a plate cut in thirds with potatoes, meat and vegetables *[typical Dutch meal]*, then it won’t be successful. Not in our culture.” (Turkish male)

Preferences regarding the advisor’s ethnicity varied among respondents in the quantitative study. About half of the Turkish respondents (55 % (95 % CI 48;63)) and of the Moroccan respondents (46 % (95 % CI 38;54)) found it a little to very important that the person that provides information has the same ethnicity as the recipient (see Fig. 1). An advantage of ethnicity-matched professionals is, as stated in the qualitative data, that a person speaks the same language.

“Of Turkish origin is easier, right? Then you understand more, so you will know more. You get more information, right?” (Turkish female)

Some participants in the qualitative studies were, however, sceptical towards a person from their own cultural background, because they were afraid of gossip within the community.

“At the end of the day, they rather prefer not to have a Moroccan counsellor, because they don’t want to air their dirty laundry in public.” (Moroccan male)

Twenty-three percent (95 % CI 16;29) of the Turkish respondents and 29 % (95 % CI 22;36) of the Moroccan respondents found it a little to very important that the information provider was of the same gender. In particular, some female participants in the qualitative studies explained why gender-matching is important. The following quotes illustrate that some women would be hesitant to talk with a male advisor and would feel better understood by a female advisor.

Woman 1: “We think women are always better. Women are also more sociable.”

Woman 2: “Being embarrassed for men. Can’t say everything.”

Woman 1: “Women do understand each other better than men.” (Turkish females)

#### Language

The preferred language for information materials differed between participants of Turkish and Moroccan origin (Chi-Square = 34.8, *p* = 0.000). Twenty-nine percent (95 % CI 22;36) of the Turkish respondents and 25 % (95 % CI 18;32) of the Moroccan respondents did not have a preference about receiving information materials in either the local language or their native language. The majority of Turkish respondents, however, wanted to receive information in Turkish (61 % (95 % CI 53;69)). Among the Moroccan respondents, the preferred language was rather divided: 37 % (95 % CI 29;45) preferred their native language, whereas 38 % (95 % CI 30;46) preferred the local language. In the qualitative data, many Turkish and Moroccan participants often stated that they would prefer to get the advice in their mother tongue. A reason why they preferred advice in their mother tongue was that they needed it in order to understand the advice better, as they might be less fluent in the local language than in their mother tongue.

Woman 1: “In their own language it is easier, yes.”

Woman 2: “They will also take it more seriously, because they hear it themselves, not via another, no, directly.” (Moroccan female adult children)

## Discussion

This current mixed-methods study gave valuable insights into what might be needed in order to reach individuals of Turkish and Moroccan origin in the Netherlands for two different activities: a health check and (lifestyle) advice. Although health checks and lifestyle advice are different activities, some common strategies could be identified to increase the reach among Turkish and Moroccan immigrants for preventive health services. The results of this study suggest that the GP may be a promising contact in order to reach these groups and that possible language barriers should be addressed. Table 6 gives an overview of the strategies identified specifically for health checks and lifestyle advice.

**Table 6:** Overview of explored strategies to reach Turkish and Moroccan immigrants for preventive health services

Health check	
□	*By whom*: Invitation by GP or – mainly in case of Turkish migrants – by a medical specialist
□	*Where*: At the GP’s office or at home, on paper
□	*Language*: Provide invitation and questionnaire in both the local language and mother tongue
Lifestyle advice	
□	*How*: Consider whether the topic is suitable to discuss in a group or should be discussed one-on-one
□	*By whom*: A physician/doctor or someone professional in that field, who is also familiar with the target groups’ (eating) behaviour
□	*Language*: Provide information in both the local language and mother tongue

The GP was indicated as the most preferred source for both the invitation for the health check and advice about CVD. Involving the GP may be a promising strategy to reach individuals of Turkish and Moroccan origin given that these groups often visit their GP [20], and GPs in the Netherlands, in general, have a positive attitude towards primary prevention of cardio-metabolic diseases [27]. However, providing preventive care might not be self-evident in all general practices, and some GPs might consider it a task for other health professionals [28].

A low proficiency in the local language was often used to explain the target groups’ preference. A lack of local language skills has been identified as one of the major barriers in reaching minorities for healthcare services [29]. The participants in this mixed-methods study discussed several strategies to overcome language barriers, like offering translated information materials or involving ethnically matched professionals. Although the involvement of ethnically matched professionals can help to overcome language barriers, our participants explained, as also reported in the literature [30], that fear of gossip can be a reason to prefer Dutch professionals over ethnically matched professionals.

In general, the Turkish and Moroccan respondents shared similar preferences. However, it was the respondents of Turkish origin rather than those of Moroccan origin that preferred to be invited for a health check by a medical specialist/hospital. Another notable difference between the two ethnic groups could be seen for preferred language. The preference for information materials in their mother tongue was more prominent among the Turkish respondents than among the Moroccan respondents. This could possibly be explained by differences in proficiency in the local language. Persons with a lower proficiency in the local language find it rather important that leaflets are provided in their mother tongue [31]. In the Netherlands, persons of Turkish origin more often have difficulties with reading Dutch than persons of Moroccan origin [32].

Overall, the findings of this study are in line with previously suggested strategies to reach ethnic minorities for preventive health services [33–35]. Bell and colleagues, for example, concluded that translated information materials and a GP endorsement letter were beneficial in recruiting ethnic minorities for breast screening [33]. An added value of our study is that a mixed-methods approach was used to research how the target groups want to be reached for preventive health services. It was therefore possible not only to quantitatively identify the target groups’ preferences, but also to qualitatively explain their preferences, and this helps us to better understand why these specific strategies are necessary. This study identified promising strategies for health professionals how to reach an underrepresented group for preventive healthcare services. It is, however, important to find out how feasible it is to meet the target groups’ preferences in practice, as for example time and financial constraints could play a role in the implementation of these strategies.

The current study focused mainly on the preferred source, location and language required to reach the target groups. However, in relation to health checks or lifestyle advice, as stated by some participants in this study, it is also important that the content suits the needs and behaviours of the targeted individuals. Therefore, in our efforts to effectively reach these groups, it is also necessary to get insight into the target groups’ preferences regarding the content of health checks or lifestyle advice.

Some methodological concerns should be taken into consideration regarding the interpretation of the results. Although a representative sample design was used to recruit respondents for the quantitative study, more Moroccan men than women participated in it. Therefore, it could be argued that the answers are not representative of the general 45+ Moroccan population in the Netherlands. However, differences between the answers of the Moroccan men and women were small and weighing the data for gender did not change the results substantially (data not shown).

The current study used existing data, which is an advantage, as mixed methods research can be expensive and time consuming. A disadvantage is that the four studies were not designed for answering the research questions of the current study. As a consequence, the topics and study population of the four original studies were not completely comparable. For example regarding advice, the quantitative study focused mainly on advice regarding CVD, whereas the qualitative studies focused on advice regarding healthy eating and physical activity. CVD is a medical condition, and this might explain why the quantitative data merely showed that the GP should give the advice. It could be that, for lifestyle advice specifically, other information sources are preferred. From the qualitative data, it appeared that it is at least important that the information source for lifestyle advice is someone professional or specialised.

The persons in the quantitative study were partly selected via a database consisting of persons who participate in research fairly regularly. It can be speculated that persons who are used to participating in research, especially in the case of questionnaires, are more likely to have higher literacy skills in the local language. As a consequence, in the quantitative study, the preference for information materials in one’s native language and the importance of ethnicity matching in order to overcome language barriers might be under-recognised.

In the mixed-methods approach, it was chosen to give a quantitative priority, meaning that greater emphasis was placed on the quantitative findings for answering the study question. The qualitative data were used to explain and elaborate on the quantitative findings. As the quantitative data were leading in the analysis, one might have missed valuable qualitative data along the way. In the quantitative study, participants were limited to the given answer categories. As a consequence, it could be, for example, that other sources beside the GP are important for the target group, but were missed in the current study as they were not present in the posed answer categories of the quantitative study.

## Conclusions

This study gave important insights into preferences of adults of Turkish and Moroccan origin relating to health checks and lifestyle advice, and reveals some promising strategies to reach these ethnic minorities for preventive healthcare services. The GP is considered as a promising contact to reach Turkish and Moroccan adults in the Netherlands for health checks and lifestyle advice. It might be necessary to provide information in individuals’ native language to overcome language barriers. In addition, the content of (lifestyle) advice must be tailored. The obtained insight into the preferences of Turkish and Moroccan adults regarding reach for preventive healthcare services could help professionals to successfully target these groups. It is important to find out how feasible it is to meet the target groups’ preferences in practice.
